# Effectiveness of an individualised treatment plan compared with a standard exercise programme in women with late-term shoulder impairments after primary breast cancer treatment: a randomised controlled trial

**DOI:** 10.2340/1651-226X.2025.42737

**Published:** 2025-03-19

**Authors:** Kim M. Feder, Marianne D. Lautrup, Sabrina M. Nielsen, Heidi K. Egebæk, Hans B. Rahr, Robin Christensen, Kim G. Ingwersen

**Affiliations:** a Department of Physiotherapy, Vejle Hospital, University Hospital of Southern Denmark, Vejle, Denmark; b Department of Regional Health Research, University of Southern Denmark, Odense, Denmark; c Department for Applied Research and Development, University College South Denmark (UC SYD), Esbjerg Ø, Denmark; d Section for Biostatistics and Evidence-Based Research, the Parker Institute, Bispebjerg and Frederiksberg, Hospital, Frederiksberg, Denmark; e OPEN – Open Patient data Explorative Network, Odense University Hospital, University of Southern Denmark, Odense C, Denmark; f Department of Surgery, Vejle Hospital, University Hospital of Southern Denmark, Vejle, Denmark; g Department of Plastic and Breast Surgery, Aarhus University Hospital, Aarhus N, Denmark; h Research Unit of Rheumatology, Department of Clinical Research, University of Southern Denmark, Odense University Hospital, Odense C, Denmark

**Keywords:** Survivorship, late side effects, rehabilitation, physical therapy

## Abstract

**Background and purpose:**

Research focuses on lymphedema, yet up to 50% face chronic shoulder issues 6 years post-treatment, while rehabilitation for this group is unclear. This trial aimed to assess the clinical effects of a shoulder expert assessment followed by an individualised treatment plan (Intervention Group; IG) compared with standardised exercises delivered as a pamphlet (Control comparator Group; CG), on changes in Shoulder Pain and Disability Index (SPADI) from baseline to week 12.

**Material and methods:**

This 12-week, assessor-blinded, parallel-group randomised controlled trial included women with late-term shoulder impairments 3–7 years post-breast cancer. Participants were randomized (1:1 allocation) and stratified by surgery and radiotherapy. Outcomes were assessed at baseline, 4, 8, and 12 weeks. Primary endpoint was SPADI overall score change at 12 weeks, analysed using a mixed model. The trial was designed to detect a between-group difference of 8 points on SPADI overall score after 12 weeks. Secondary outcomes were SPADI pain/function, global perceived effect, changes in shoulder pain numeric rating scale, active and passive shoulder range of motion.

**Results:**

After 12-weeks, no between-group difference in SPADI was found between IG and CG (−10.5 and −14.4, respectively), corresponding to a difference of −3.9 points (95% CI −11.9 to 4.1; P = 0.34).

**Interpretation:**

The effects on shoulder pain and disability symptoms of a shoulder expert assessment followed by an individualised treatment plan was not superior to standardised exercises delivered as a pamphlet in women with late-term shoulder impairments 3–7 years post-breast cancer.

**Trial registration:**

ClinicalTrials.gov (NCT05277909).

## Introduction

The most common cancer among women globally is breast cancer, with nearly 2.3 million new cases yearly in 2022 [1]. In Denmark, roughly 5,000 women are diagnosed with breast cancer each year [2]. Thanks to early detection and improved surgical and oncological treatments [3], 5-year survival rate is 90% [4]. Standard surgical treatment is mastectomy or breast-conserving surgery (BCS) in combination with sentinel lymph node biopsy (SLNB) or axillary lymph node dissection (ALND] [3]. To reduce the risk of relapse and improve long-term survival, women with breast cancer can also receive radiotherapy [3].

However late-term impairments (e.g. persistent or long-lasting health problems that develop as a delayed consequence of breast cancer treatment) remain common [5, 6], particularly lymphedema, neuropathy, or disability symptoms like impaired shoulder function and shoulder pain [5–7]. These impairments increase the risk of depression, anxiety [8], and decrease quality of life [5]. Research has mainly focussed on arm lymphedema over other upper limb impairments [6, 7]. However, up to 50% of patients experience chronic shoulder problems 6 years after surgery [5–7, 9, 10], but optimal rehabilitation for this group remains unclear [6, 11, 12], leaving a significant knowledge gap. With an age of ≤ 62 years at diagnosis [13], many remain active in the workforce, making it vital to improve shoulder management after treatment for patient and societal benefits.

Rehabilitation needs of these women, are considered complex and diverse depending on type of surgery, oncological treatment, the individual pathophysiological response to treatment and its potential complications. Therefore, individualisation is considered fundamental for optimal rehabilitation [11, 12]. At present, evidence-based guidelines for individualised rehabilitation and referral of patients for advanced rehabilitation are lacking [12, 14].

While surgical procedures, like axillary interventions, affect shoulder function and neuromuscular tissues [9], radiotherapy further damages the shoulder by causing muscle fibrosis and stiffness, impairing muscle function [9, 10, 15]. To some degree, these late-term impairments of the shoulder after breast cancer treatment, can resemble impairments, following traumatic or degenerative mechanism [16], among ordinary shoulder. To increase success of implementation of organisational pathways for referral and treatment, one can benefit from utilising existing treatment options. Therefore, utilising existing treatment paths with extensive knowledge of shoulder impairments, the interventions in this study were based on established treatment paths in Denmark. Patients in Denmark with persistent shoulder complaints, are typically referred to a specialised orthopaedic department for example at Vejle Hospital, where they receive a shoulder expert assessment at the hospital followed by an individually tailored treatment plan (e.g. specific exercise instructions performed at home or supplemented by supervise sessions at the hospital, municipality or private practice; corticosteroid injections in the shoulder; surgery).

The objective of this study was to assess the clinical effects on shoulder pain and disability symptoms of a shoulder expert assessment followed by an individualised treatment plan (*Intervention Group; IG*), compared with standardised exercises delivered as a pamphlet (*Control comparator Group; CG*) in women with late-term shoulder impairments 3–7 years after primary breast cancer treatment. We hypothesise that women allocated to *IG* would experience improvements in shoulder function and pain superior to those allocated to *CG,* which will include a between-group difference of ≥ 8 points ([minimal important difference (MID)] = ≥ 8 points) on Shoulder Pain and Disability Index (SPADI) overall score after 12 weeks.

## Material and methods

### Study design

This study was a stratified (by type of surgery and radiotherapy), parallel-group, assessor-blinded, randomised (1:1 allocation), controlled trial conducted in Denmark from the 4th of April to the 10th of October 2022. Primary and key secondary outcomes were collected at baseline, 4, 8, and 12 weeks. Primary endpoint was the change in SPADI score from baseline to 12 weeks follow-up. Details are published in the study protocol [17].

### Participants

We recruited women with primary breast cancer who underwent unilateral BCS or mastectomy between 2015 and 2019, through a cross-sectional survey regarding late-term upper limb impairments 3–7 years after primary breast cancer treatment. To be eligible, women had to: (1) score ≥ 15 on the Disabilities of the Arm, Shoulder and Hand (Quick DASH) [18]; (2) live within a radius of 75 km from Vejle Hospital, Denmark; (3) be between 18 and 71 years at the time of surgery; and (4) agree to participate by signing a written informed consent form.

Women were excluded if they had a cancer relapse and received new treatment or had undergone breast reconstruction at any time, due to the risk of double exposure leading to late-term impairments. Further exclusion criteria were: severe lymphedema (an average score ≥ 70% in the first seven items on LYMPH-ICF-DK) [19], bilateral breast cancer surgery, previous surgery or fractures in the affected shoulder, currently receiving chemo-, immuno- or radiotherapy, co-morbidities expected to influence shoulder function (e.g. rheumatoid arthritis, previous stroke, multiple sclerosis) or other reasons for exclusion (e.g. pregnancy, not legally competent or unable to comprehend information) [17].

### Setting and locations

This trial was conducted at Department of Physio- and Occupational Therapy and Orthopaedic Department, Vejle Hospital, Denmark. The study recruited eligible participants from a nationwide cross-sectional questionnaire study among women treated for breast cancer within 3–7 years. Eligible participants were informed about the trial through the questionnaire with the option of receiving further information, where after they were informed by phone. Hereafter, detailed written information was sent via e-Boks, and participants were contacted again for their final decision.

### Interventions

Participants randomised to *IG* were referred to a shoulder expert assessment, performed by one of four experienced specialists (surgeons and physiotherapists) with 6+ years’ experience in diagnosing shoulder disorders, in the specialised orthopaedic department at Vejle Hospital. Assessments included shoulder-specific examination including history, standard clinical tests [20], including X-ray and ultrasonography. The results guided the individualised treatment plan, and it was up to the specialist to decide treatment through shared decision-making with the patient. In general, the individualised treatment plan included referral to physiotherapeutic treatment at hospital, municipality or private practice, if relevant, combined with an ultrasound-guided corticosteroid injection and/or advice in management of shoulder load in daily life. A typical physiotherapeutic treatment included manual treatments (e.g. myofascial therapy, ischemic compression of trigger points and tissue displacement) and shoulder exercises specific for the rotator cuff. The type of shoulder exercises (e.g. strength, resistance or cardio), trained muscles, and frequency per week were individualised, whether at hospital, municipality or private practice (Supplementary Figure 1). This intervention is a pragmatic package solution in which we aim to test the effect of what is examined and treated within the hospital regimen. Therefore, *IG* participants were advised not to participate in any concomitant treatment parallel with the intervention.

Participants allocated to *CG* was given a pamphlet without verbal instructions. The pamphlet explained an exercise programme consisting of three mobility exercises, three stretching exercises for breast and shoulder area, one connective tissue displacement and four strengthening exercises for the rotator cuff. Patients were advised to perform mobility exercises (1 set with 5–10 repetitions), stretching exercises (1 set in 30 s) and connective tissue displacement (1 set in a few minutes) twice a day, while strength exercises were advised to be performed once a day with three sets of 12 repetitions (Supplementary Figure 2). For *CG*, any concomitant treatment was allowed during the trial [17].

### Outcome measures

#### Primary outcome

The primary outcome measure was change in SPADI overall score. The SPADI overall score is a composite score summarising questions regarding shoulder pain (questions 1–5) and shoulder function (questions 6–13) within the last week, and ranges from 0 to 100 (best to worst) [18, 21]. Shoulder Pain and Disability Index is a valid and reliable measure for use in patients with shoulder impairments [18, 21].

Primary endpoint was assessed 12 weeks after baseline. Secondary endpoints were at 4 and 8 weeks after initiating the treatment. At baseline and 12 weeks follow-up, patients answered the questionnaire electronically through the Research Electronic Data Capture (REDCap) [22] in an undisturbed room at Vejle Hospital. Questionnaires at 4 and 8 weeks were sent electronically to the patients’ personal electronic mail-box (e-Boks), and answered at home. If a woman did not reply within 3 days, a reminder e-mail was sent, followed by contact by phone 4 days after the reminder.

#### Key secondary outcomes

Key secondary outcomes were:

- Mean change in SPADI pain 0–100 and mean change in SPADI function 0–100 (best to worst; 0, 4, 8, and 12 weeks),
- Global perceived effect (GPE) (1 = better, 2 = unaltered, 3 = worse; 4, 8, and 12 weeks),
- Mean change in maximum shoulder pain intensity, shoulder pain during general activities, shoulder pain at rest, and shoulder pain during sleep; all within the previous 24 h in the affected shoulder (Numeric Rating Scale [NRS]: 0–10; best to worst [23]; 0, 4, 8, and 12 weeks),
- Mean change in active and passive range of motion (A-ROM and P-ROM) in flexion/internal rotation/external rotation/abduction measured in degrees through a smartphone inclinometer; GetMyROM [24] (12 weeks),
- Mean change in shoulder pain during active and passive flexion/internal rotation/external rotation/abduction (NRS: 0–10; best to worst; 12 weeks).

Details of how outcome measures were introduced and assessed are available in the study protocol [17].

#### Response to treatment

The individual patient were classified as having a clinical response to treatment if the SPADI change score improved by 18 point or more [25].

### Sample size and power considerations

To achieve a statistical power of at least 85% with a two-sided significance level of α = 0.05 and an anticipated standard deviation (SD) of 15.41 SPADI points [26], the estimated total sample size was *n* = 130 (~ 65 participants in each group), enabling detection of a target between-group difference of 8 points on SPADI overall score [25].

### Randomisation, allocation concealment, and blinding

Participants were randomised to *IG* or *CG* with a 1:1 allocation ratio, using a computer-generated randomisation sequence on permuted blocks of 2 to 6, and stratification by type of surgery and radiotherapy. Randomisation sequence was generated by an independent data manager and uploaded into REDCap [22], securing concealed allocation. An independent secretary performed the randomisation, referred participants for examination in the specialised orthopaedic department at Vejle Hospital (*IG*) or delivered the pamphlet to *CG,* and booked an appointment for follow-up measurements for all participants 12 weeks after initiating the treatment.

The primary investigator (KMF) performed all baseline and follow-up assessments, and was blinded toward group allocation. Before follow-up, the primary investigator strongly encouraged (both written and oral) participants not to disclose their allocated treatment. Due to the study design participants, the independent secretary, orthopaedic specialists, and physiotherapists who carried out the individualised treatment could not be blinded to treatment allocation. Participants were not aware of the hypothesis being tested, and none of the orthopaedic specialists or the independent secretary participated in data analysis or preparation of the manuscript. The participants were told the aim of the study was to investigate which intervention is better (*IG* or *CG*), but not that we favour one over the other. An independent biostatistician who performed the primary and key secondary outcome analyses was blinded to treatment allocation. Based on blinded results from the Intention-To-Treat (ITT) analyses, an interpretation was written and made publicly available [27] by the trial group (March 15, 2023), prior to the disclosure of the group identities [28]. After the disclosure of group allocation, the independent biostatistician conducted the prespecified sensitivity analysis and evaluation of serious adverse events (SAEs) [17].

### Statistical methods

The statistical analysis plan (SAP) [29] was made publicly available before data extraction. Main analyses were based on the ITT population, which included all participants enrolled and randomised [30, 31]. Continuous outcomes were analysed as the change from baseline with the use of repeated measures mixed-effects linear models with the patient identification number as a random effect and with the baseline score, treatment group (*IG* or *CC*), time (baseline, 4, 8 and 12 weeks), type of surgery and radiotherapy, and interactions between treatment groups and time as fixed-effect factors [29]. Within-group changes from baseline are reported as least squares means (LS Means) and standard errors (SEs) or median (Interquartile range [IQR]) depending on the data distribution of the studentised residuals. The between-group differences are presented as the difference in least squares means with 95% Confidence Intervals (CIs) (Difference in LSMeans 95% CI) or medians with approximated 95% CIs (approximated 95% CI) [30].

According to the pre-specified SAP, participants were classified as having a clinical response if the SPADI change score was 18 points or more [25, 29]; in case of missing data for the dichotomous endpoints, a non-responder assumed imputation was applied. The main analyses were based on the ITT population; missing data were handled with the use of the mixed-effects linear-models approach with an assumption that data were missing at random, in accordance with the working assumption underlying these models [30, 32]. Sensitivity analyses on SPADI scores also performed on the ITT population included a single-step non-responder imputation with replacement of missing data by the value at baseline (baseline observation carried forward, BOCF) [33]. All data analysis and estimations were performed using SAS (SAS Institute Inc., Cary, North Carolina, USA) and STATA (Statacorp, College Station, Texas, USA) software.

### Ethics and registration

The Regional Health Research Ethics Committee of Southern Denmark accepted the trial (version 3) on 15 February 2022 (Project-ID: S-20200021) and the Danish Data Protection Agency accepted on 17 May 2019 (Journal No. 19–16321), which secure the principles of the Declaration of Helsinki [34]. The authors ensure the completeness of the reported data, analyses, and compliance to the previously published trial registration on ClinicalTrials.gov (NCT05277909), the study protocol [17], and the final SAP [29].

All women provided written informed consent before participating. Participants were informed that if they were randomised to *CG* and did not experience relevant improvement, they would be offered the same treatment as *IG*, after they completed their intervention and follow-up assessment. Likewise, this applied to the opposite group.

## Results

### Characteristics of the participants

**Figure 1:**
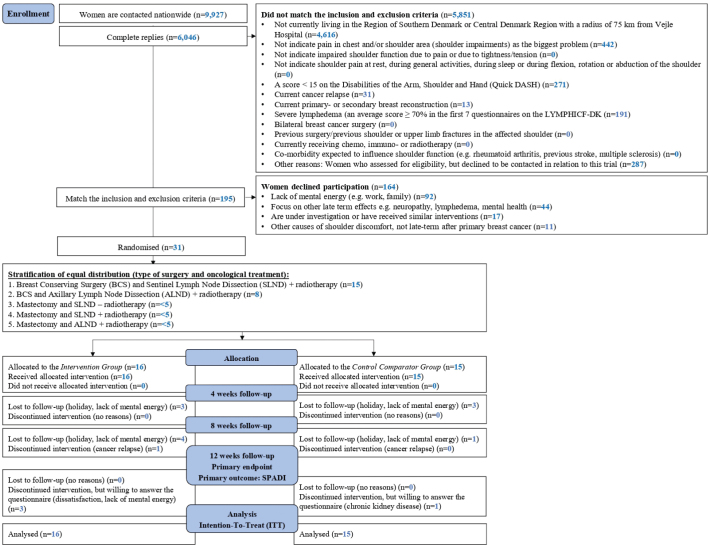
Flowchart.

Based upon the nationwide cross-sectional questionnaire to 9,927 women with a response rate of 60.9% (6,046 complete replies), 195 women were eligible for enrolment and gave consent to be contacted by phone for further information about the trial. All 195 women were contacted and 164 declined to participate (Figure 1). A total of 31 women were thus enrolled and randomised: *n* = 16 to *IG* and *n* = 15 to *CG*. Baseline characteristics for the two groups are reported in Table 1 [31].

**Table 1:** Baseline characteristics in the ITT population.*

	Intervention group (IG) (*N* = 16)	Control comparator group (CG) (*N* = 15)	Total combined (*N* = 31)
**General characteristics**			
Age – years	54.4 (10.9)	57.6 (9.8)	56.0 (10.3)
Height – cm	165.5 (4.3)	164.3 (6.2)	164.9 (5.2)
Weight – kg	78.2 (14.4)	77.9 (15.0)	78.0 (14.5)
Body Mass Index – kg/m^2^	28.5 (5.1)	28.9 (5.6)	28.7 (5.2)
**Alcohol consumption**			
0 units per week – no. (%)	6 (37.5)	6 (40.0)	12 (38.7)
1–7 units per week – no. (%)	10 (62.5)	9 (60.0)	19 (61.3)
8–14 units per week – no. (%)	0 (0)	0 (0)	0 (0)
≥ 15 units per week – no. (%)	0 (0)	0 (0)	0 (0)
**Smoking habits**			
Smoker – no. (%)	0 (0)	0 (0)	0 (0)
Current smoker – no. (%)	6 (37.5)	8 (53.3)	14 (45.2)
Not a smoker – no. (%)	10 (62.5)	7 (46.7)	17 (54.8)
**Highest education level**			
Short – no. (%)	10 (62.5)	10 (66.7)	20 (64.5)
Long – no. (%)	6 (37.5)	5 (33.3)	11 (35.5)
**Employment**			
Employed for wages or self-employed – no. (%)	11 (68.7)	7 (46.7)	18 (58.0)
Sick leave – no. (%)	0 (0)	0 (0)	0 (0)
Retired – no. (%)	5 (31.3)	8 (53.3)	13 (42.0)
**Index shoulder (the affected shoulder)**			
Right side – no. (%)	9 (56.3)	7 (46.7)	16 (51.6)
Left side – no. (%)	7 (43.7)	8 (53.3)	15 (48.4)
**Dominant side affected**			
Yes – no. (%)	9 (56.3)	6 (40.0)	15 (48.4)
No – no. (%)	7 (43.7)	9 (60.0)	16 (51.6)
Mean duration of shoulder symptoms – months	5.6 (1.1)	5.7 (1.6)	5.7 (1.4)
**Outcome measures**			
SPADI overall score^†^ – 0 to 100	45.7 (19.4)	47.6 (16.2)	46.6 (17.7)
SPADI shoulder pain – 0 to 100	53.4 (19.5)	58.4 (12.7)	55.8 (16.5)
SPADI shoulder function – 0 to 100	38.0 (21.7)	36.8 (23.4)	37.5 (22.2)
NRS maximum shoulder pain intensity – 0 to 10	6.4 (1.5)	6.4 (1.3)	6.4 (1.4)
NRS shoulder pain during general activities – 0 to 10	4.6 (2.6)	4.2 (1.7)	4.4 (2.2)
NRS shoulder pain at rest – 0 to 10	3.8 (2.6)	3.7 (1.7)	3.7 (2.2)
NRS shoulder pain during sleep – 0 to 10	4.0 (2.9)	4.5 (2.8)	4.2 (2.8)
**P-ROM in the affected shoulder – degree**			
Flexion	118.2 (18.1)	124.1 (18.5)	121.1 (18.2)
Internal rotation	53.4 (19.0)	66.3 (15.6)	59.6 (18.3)
External rotation	36.0 (16.1)	51.3 (22.1)	43.4 (20.4)
Abduction	83.9 (31.2)	107.2 (30.0)	95.2 (32.4)
**NRS passive shoulder pain during – 0 to 10**			
Flexion	4.3 (2.4)	3.9 (1.8)	4.1 (2.1)
Internal rotation	4.1 (2.5)	2.3 (2.0)	3.3 (2.4)
External rotation	4.6 (2.7)	3.9 (2.4)	4.3 (2.6)
Abduction	4.8 (2.5)	4.6 (1.6)	4.7 (2.1)
**A-ROM in the affected shoulder – degree**			
Flexion	110.0 (21.9)	117.6 (16.0)	113.7 (19.3)
Internal rotation	53.5 (25.1)	68.9 (17.9)	60.9 (22.9)
External rotation	34.0 (22.0)	48.7 (26.5)	41.1 (25.0)
Abduction	78.2 (29.5)	98.9 (32.3)	88.2 (32.1)
**NRS active shoulder pain during – 0 to 10**			
Flexion	4.5 (2.2)	3.9 (1.8)	4.2 (2.0)
Internal rotation	4.1 (2.6)	2.3 (2.1)	3.2 (2.5)
External rotation	4.8 (2.9)	4.0 (2.6)	4.4 (2.7)
Abduction	4.8 (2.4)	4.8 (2.1)	4.8 (2.2)

SPADI: Shoulder Pain and Disability Index; NRS: Numeric Rating Scale; ROM: Range Of Motion.

* Values are reported as means and standard deviations (SDs) unless otherwise stated.

† SPADI ranges from 0 (best) to 100 (worst), with lower scores indicating better disease status.

Supplementary Table 1 gives an insight into what women allocated to *IG* received. Five participants referred to private practice, declined further treatments due to self-payment.

### Missing data, withdrawals and crossovers

All baseline data were acquired. At 4 and 8 weeks, six women (*IG* = 3; *CG* = 3) and (*IG* = 5; *CG* = 1) did not answer the questionnaire. The most common reason was due to summer holidays or lack of mental energy, while one withdrew at 8 weeks due to cancer relapse. At 12 weeks follow-up, all participants answered the questionnaire, but four women (*IG* = 3; *CG* = 1) did not participate in the physical tests (Figure 1). There was no crossover, and none of the participants participated in any other concomitant treatment during the trial.

### Primary outcome

Mean change in SPADI overall score was −10.5 (SE: 2.8) in *IG* and −14.4 (SE: 2.9) in *CG* from baseline to 12 weeks follow-up. For the between-group comparison, there was no difference between groups: −3.9 points (95% CI: −11.9 to 4.1; *P* = 0.34) (Table 2).

**Table 2:** Primary and key secondary outcomes at 12 weeks in the ITT population.*

Outcome	12 weeks after initiating the treatment		Between-group difference in mean improvement	
	Intervention group (IG)	Control comparator group (CG)	Difference in LSMeans (95% CI)	*P*
	LS means (SE)	LS means (SE)		
**Primary endpoint**				
Change SPADI overall score^†^ (0 to 100)	−10.5 (2.8)		−3.9 (−11.9 to 4.1)	0.34
**Key secondary outcome measures** −14.4 (2.9)				
Change SPADI pain^†^ (0–100)	−13.2 (3.9)	−16.7 (3.9)	−3.5 (−14.6 to 7.6)	0.53
Change SPADI function^†^ (0–100)	−7.8 (2.5)	−11.8 (2.5)	−4.0 (−11.0 to 3.1)	0.26
GPE impression of the treatment success (median^‡^)	2.0 [2.0; 2.0]	1.0 [1.0; 2.0]	−1.0 (−1.8 to −0.2)	0.01
Change NRS maximum shoulder pain intensity (0–10)	5.1 (0.4)	4.8 (0.4)	−0.3 (−1.4 to 0.8)	0.56
Change NRS shoulder pain during general activities (0–10)	3.5 (0.4)	3.4 (0.4)	−0.1 (−1.2 to 1.1)	0.91
Change NRS shoulder pain at rest (0–10)	2.4 (0.4)	2.0 (0.4)	−0.4 (−1.5 to 0.8)	0.56
Change NRS shoulder pain during sleep (0–10)	3.5 (0.4)	2.7 (0.5)	−0.8 (−2.1 to 0.4)	0.19
Number of treatments due to shoulder symptom (median^‡^)	0.0 [0.0; 0.0]	0.0 [0.0; 0.0]	0.0 (0.0 to 0.0)	0.00
***P-ROM in the affected shoulder (degree)***				
Flexion (median^‡^)	124.8 [107.17; 141.67]	147.7 [132.67; 154.33]	22.9 (4.38 to 41.29)	0.02
Internal rotation (median^‡^)	79.5 [62.17; 85.83]	73.3 [62.33; 85.67]	−6.2 (−20.48 to 8.15)	0.40
External rotation (median^‡^)	51.5 [36.33; 65.33]	60.5 [45.00; 74.67]	9.0 (−4.49 to 22.49)	0.19
Abduction (median^‡^)	101.8 [90.33; 114.50]	142.3 [113.00; 144.67]	40.5 (6.77 to 74.23)	0.02
***Passive NRS shoulder pain assessment during (0–10)***				
Flexion (median^‡^)	3.5 [2.0; 4.0]	2.0 [1.0; 6.0]	−1.5 (−3.8 to 0.8)	0.19
Internal rotation (median^‡^)	1.0 [0.5; 2.5]	1.5 [0.0; 3.0]	0.5 (−0.8 to 1.8)	0.45
External rotation (median^‡^)	2.0 [1.0; 4.5]	2.0 [0.0; 5.0]	0.0 (0.0 to 0.0)	0.35
Abduction (median^‡^)	4.5 [2.0; 5.5]	2.5 [1.0; 5.0]	−2.0 (−5.0 to 1.0)	0.20
***A-ROM in the affected shoulder (degree)***				
Flexion (median^‡^)	117.7 [94.83; 136.83]	136.0 [129.33; 145.67]	18.3 (−0.43 to 37.09)	0.06
Internal rotation (median^‡^)	78.7 [65.17; 84.17]	74.8 [59.67; 87.33]	−3.9 (−12.20 to 4.53)	0.37
External rotation (median^‡^)	51.0 [33.67; 69.17]	62.7 [45.00; 75.00]	11.7 (−6.10 to 29.43)	0.20
Abduction (median^‡^)	93.7 [82.67; 109.33]	138.2 [99.67; 141.67]	44.5 (5.91 to 83.09)	0.02
***Active NRS shoulder pain assessment during (0–10)***				
Flexion (median^‡^)	2.0 [2.0; 5.0]	2.0 [1.0; 6.0]	0.0 (0.0 to 0.0)	0.32
Internal rotation (median^‡^)	1.0 [0.5; 2.5]	1.5 [0.0; 3.0]	0.5 (−0.7 to 1.7)	0.41
External rotation (median^‡^)	2.0 [1.5; 4.5]	2.0 [0.0; 5.0]	0.0 (0.0 to 0.0)	0.32
Abduction (median^‡^)	4.5 [2.0; 5.5]	2.5 [1.0; 5.0]	−2.0 (−5.1 to 1.1)	0.21
**Response to treatment**				
Change SPADI clinical response^§^	31%	27%	0.9^¶^ (0.27/0.31)	

CI: confidence interval; SE: standard error; NRS: Numeric Rating Scale; SPADI: Shoulder Pain and Disability Index; ROM: Range Of Motion.

* All analyses will be based on the Intention-To-Treat (ITT) population: Using a mixed model for repeated measurements (with a mixed-effects linear-models approach for missing data); Estimates will be least squares means (LSMeans) and SE with the difference between groups reported with 95% CIs.

† SPADI ranges from 0 (best) to 100 (worst), with lower scores indicating better disease status.

‡ Median (IQR; Interquartile Range) and median differences with 95% CIs reported for these outcomes.

§ Patients classified as having a clinical response if the SPADI change score improves by 18 points or more.

¶ Risk ratio (RR) difference reported for these outcome.

### Key secondary outcomes

- For SPADI pain and SPADI function, no significant differences between *IG* and *CG* were observed at 12 weeks follow-up, with mean between-group change differences of −3.5 points (95% CI: −14.6 to 7.6; *P* = 0.53) for SPADI pain and −4.0 points (95% CI: −11.0 to 3.1; *P* = 0.26) for SPADI function.
- For *CG*, statistically significant greater improvements in GPE (impression of the treatment success) compared with *IG* at 12 weeks follow-up were observed, with a median between-group difference of −1.0 points (95% CI: −1.8 to −0.2; *P* = 0.01).
- In all pain NRS measurements, no significant differences between *IG* and *CG* at 12 weeks follow-up were observed.
- For *CG*, statistically greater improvements in P-ROM during flexion and abduction, and A-ROM during abduction were observed, compared with *IG*. The median between-group differences were 22.9 degrees (95% CI: 4.38 to 41.29; *P* = 0.02) in P-ROM flexion, 40.5 degrees (95% CI: 6.77 to 74.23; *P* = 0.02) for P-ROM abduction, and 44.5 degrees (5.91 to 83.09; *P* = 0.02) for A-ROM abduction.
- For all other active and passive movements as well as for all NRS, no statistically significant differences were found between *CG* and *IG* (Table 2).

#### Response to treatment

While the mean change was in favour of *CG*, the proportion of women who improved by 18 points or more ([clinical response] = 18 points) [24] in SPADI was 31% in *IG* and 27% in *CG*, corresponding to a risk ratio (RR) difference of 0.9 (0.27/0.31) (Table 2).

### Sensitivity analyses

Due to the few included participants in this trial and the difficulties to examine the robustness by revealing similar results, the sensitivity analyses were only performed on SPADI scores at 12 weeks in the ITT population by using a single-step non-responder imputation and presented similar results as the primary analysis (Supplementary Table 2).

### Adverse events

Two adverse events (SAEs) were recorded as serious requiring hospitalisation (breast cancer relapse in *IG* and chronic kidney disease in *CG*). This SAEs were collected from both the patient-reported questionnaire and from the medical record review conducted at the 12-week follow-up [17]. The woman with chronic kidney disease completed the treatment in this trial.

## Discussion

### Main findings

This is the first trial to compare an individualised treatment approach with a standardised exercise approach in women with late-term shoulder impairments after primary breast cancer treatment. The main finding was no significant difference (*P* = 0.34) between the individualised or standardised exercise approaches when measured by SPADI after 12 weeks. Some key secondary outcomes was statistically significant as GPE, P-ROM flexion, P-ROM abduction, and A-ROM abduction; but since the trial is underpowered it is inconclusive.

### Generalisability

In general, there is a lack of studies in the specific area of ‘late-term shoulder impairments’ among breast cancer survivors. Several trials have reported that early physiotherapeutic exercises were effective in preventing postoperative shoulder pain and impaired shoulder function within 1 year after breast cancer treatment [6, 11, 35]. However, previous trials evaluating interventions on late-term effects focus on other disabilities than shoulder pain, particularly lymphedema [5–7], leaving no data for direct comparison.

Among breast cancer survivors, mechanisms causing shoulder disorders is multifactorial. Scar tissue after surgery and radiotherapy are considered to cause tight muscle/tendon tissue or stiffness of surrounding tissue, decreased stability, reduced muscle strength, and range of motion in the shoulder joint [9, 10, 15]. Breast cancer patients undergoing more extensive breast (e.g. mastectomy) and axillary surgery and radiotherapy experience more complicated and long-term mechanical changes of the shoulder [9, 10, 15]. A recent study described that women who received exercises including individualised treatments made them feel more confident and helped them feel to have control over their well-being and diminished their fear of moving the arm/shoulder [11]. Furthermore, participants valued the therapeutic support in relation to psychological and emotional aspects [11]. Because of the complexity of the problem, an individualised treatment should be considered for all patients. In contrast, our study showed that a simple set of exercises can induce improvements in shoulder function in breast cancer patients even 3–7 years after treatment.

Socioeconomic status is also to be considered in the treatment plan. Five participants in *IG*, actively declined further treatments due to the self-payment in private practice. It is important that all women get the same access to treatments to minimise social inequalities [36]. The strength of *IG* was that it accommodated individual needs. However, a disadvantage could be that the expert shoulder assessment was conducted at Vejle Hospital, which may result in long travel distances for women living outside the region, thereby limiting equal access for all. The pamphlet in *CG* addresses this inequality, but it does not take into account disadvantaged individuals who may lack the resources to implement the pamphlet’s content.

Our underpowered results should not discourage further efforts to improve shoulder rehabilitation for breast cancer survivors.

### Strengths and limitations

This trial had some limitations. First of all, we were only able to include 31 of the expected 130 women (~24%), resulting in a statistically underpowered study. This is a critical limitation that can affect the trials ability to detect meaningful differences between the interventions. Furthermore, the low recruitment rate could raise a concern about potential selection bias towards recruiting more resourceful women from the questionnaire study [37]. This could result in recruiting more resourceful women in both groups, and hence making the result of *CG* ‘bloated’ compared to if had been possible to recruit less resourceful women. A wider recruitment could give a more nuanced picture of the population and then favouring *IG* and less resourceful women with less resources in need of more individualised support. These limitations hinder the generalisation of these results to the general patient population, and future studies should ensure better representability. In any case, what we tested here is a complex intervention, and its effect was assessed as a whole.

On the other hand, this trial was the first to compare individualised treatment with a set of standardised exercises for women with late-term shoulder impairments after primary breast cancer, which address a research gap. Furthermore the current trial demonstrated several methodological strengths, including blinding of outcome assessor, a blinded outcome analysis performed by independent biostatisticians, interpretation of the results prior to unblinding, and the publication of a detailed study protocol and a SAP prior to analysis of data [17, 29]. These practices enhance the credibility of this study.

### Implications and future research

Surprisingly, only 13% of eligible participants with shoulder impairments chose to join this trial. Possible reasons include reluctance to revisit memories of their life-threatening disease, viewing shoulder impairment as a natural consequence of treatment, or believing no treatment can help. Others have returned to busy work lives or participated in prior trials with lengthy follow-ups and prefer not to join new ones. In this study, the majority declined participation because of a lack of mental energy, and focus on other late-term factors such as lymphedema and mental health (Figure 1). Such factors should be considered in future studies.

## Conclusion

The effects on shoulder pain and disability symptoms of a shoulder expert assessment followed by an individualised treatment plan was not superior to standardised exercises delivered as a pamphlet in women with late-term shoulder impairments 3–7 years after primary breast cancer treatment.

## Supplementary Material

Effectiveness of an individualised treatment plan compared with a standard exercise programme in women with late-term shoulder impairments after primary breast cancer treatment: a randomised controlled trial

## Data Availability

Data are available on reasonable request.
