# Supplementary material for: Effectiveness of an individualised treatment plan compared with a standard exercise programme in women with late-term shoulder impairments after primary breast cancer treatment: a randomised controlled trial
Source: Acta Oncol. 2025 Mar 19;64:42737. doi: 10.2340/1651-226X.2025.42737 (PMC11971842; doi:10.2340/1651-226X.2025.42737)
Supplement: Effectiveness of an individualised treatment plan compared with a standard exercise programme in women with late-term shoulder impairments after primary breast cancer treatment: a randomised controlled trial [file AO-64-42737-s1.pdf]

Supplementary material has been published as submitted. It has not been copyedited, or typeset by Acta Oncologica

Supplementary Figure 1: The content of the *Intervention Group*

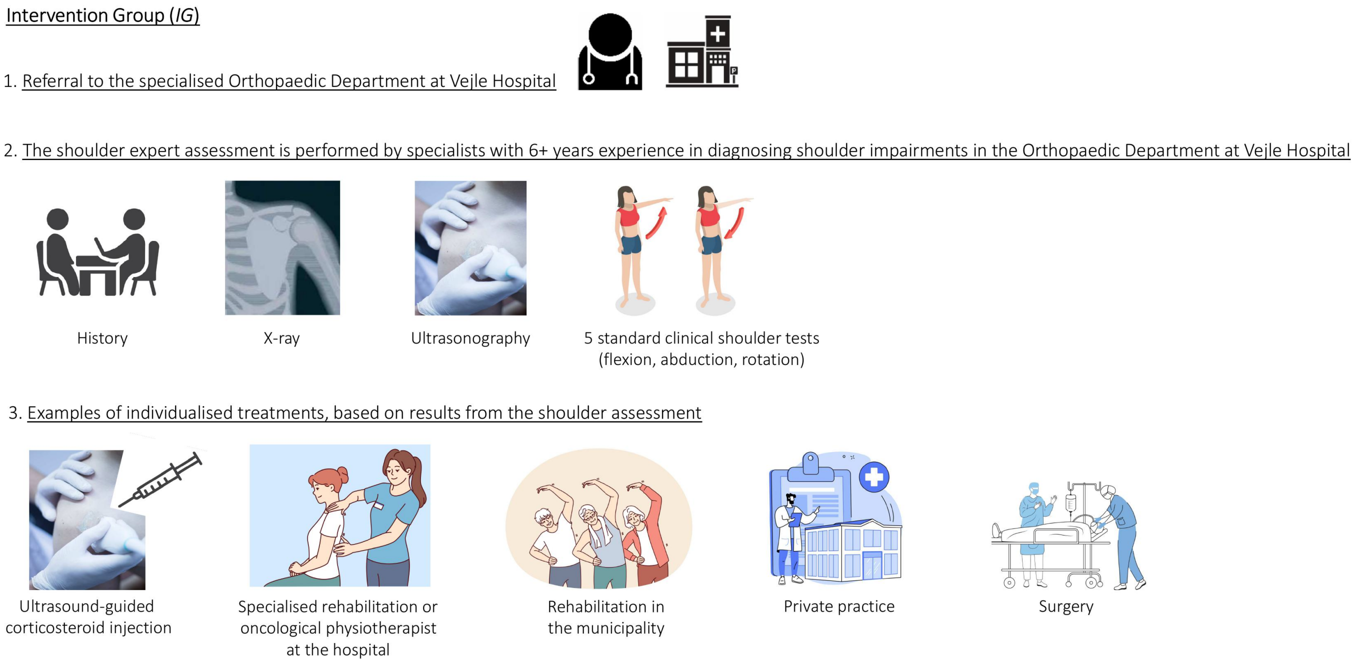

Supplementary Figure 2: The content of the *Control comparator Group*

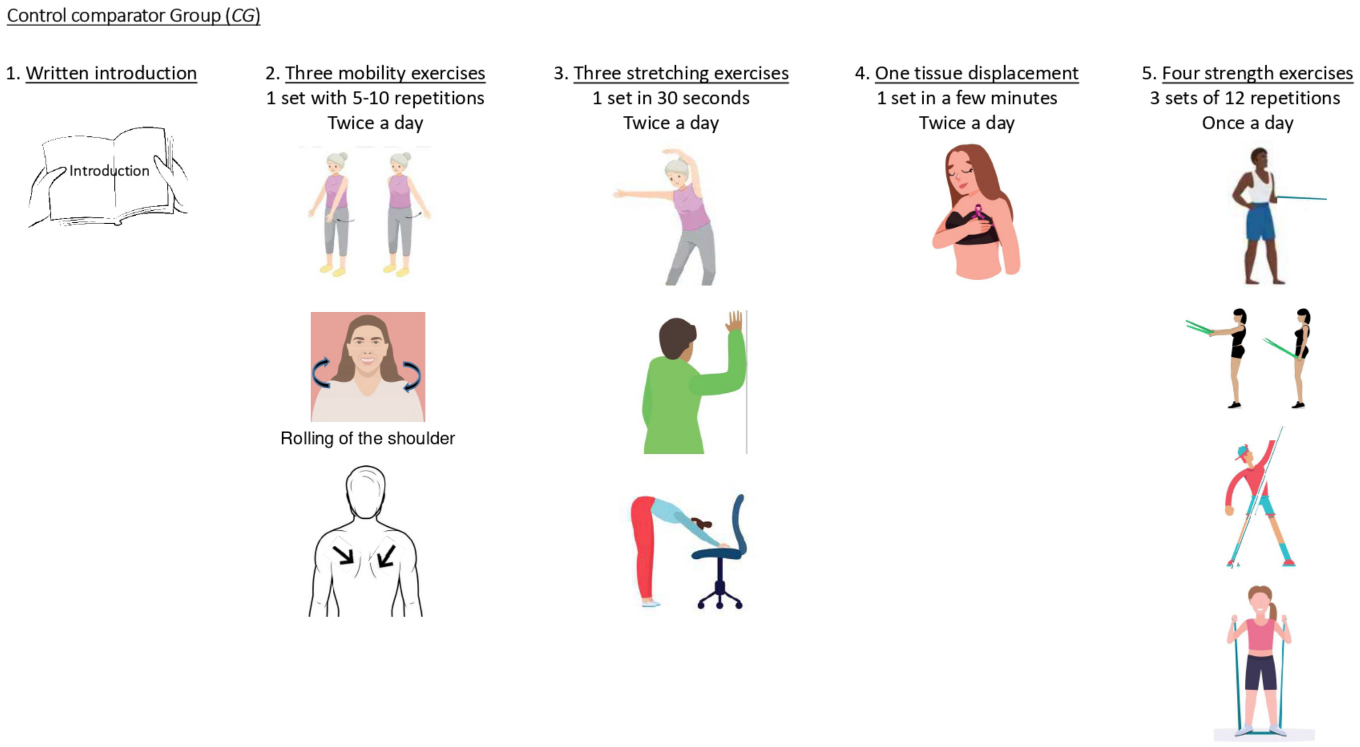

Supplementary Table 1: Description of treatments received in *IG* (N=16)

**1. Ultrasound-guided corticosteroid injection in the shoulder**<br><br>(performed at the Orthopaedic Department, Vejle Hospital)<br><br>**Physiotherapy at Vejle Hospital**<br><br>(strength training with weights/machines, elastic bands; self training after following instructions from a physiotherapist)<br><br>**Follow-up** (physical or telephone)
**2. Ultrasound-guided corticosteroid injection in the shoulder**<br><br>(performed at the Orthopaedic Department, Vejle Hospital)<br><br>**Physiotherapy at Vejle Hospital**<br><br>(strength training with weights/machines, elastic bands; self training after following instructions from a physiotherapist)<br><br>**Follow-up** (physical or telephone)<br><br>**Physiotherapist in private practice** (massage)
**3. Ultrasound-guided corticosteroid injection in the shoulder & follow-up**<br><br>(performed at the Orthopaedic Department, Vejle Hospital)<br><br>**Masseuse in private practice** (massage)
**4. Physiotherapy at Vejle Hospital** (mobility training and stretching)<br><br>**Follow-up** (physical or telephone)
**5. Referral to another professional in the municipality**<br><br>(performed at the Orthopaedic Department, Vejle Hospital)<br><br>**Physiotherapy in the municipality**<br><br>(strength training with weights/machines, elastic bands; self training after following instructions from a physiotherapist, mobility training and stretching, tape)
**6. Referral to another professional in the private practice; self-payment**<br><br>(performed at the Orthopaedic Department, Vejle Hospital)<br><br>**Physiotherapist in private practice** (group training)
**7. Referral to another professional in the private practice; self-payment**<br><br>(performed at the Orthopaedic Department, Vejle Hospital)

Supplementary Table 2: Sensitivity Analysis at 12 weeks in the ITT population using a single-step non-responder (BOCF) imputation

**Outcome**	***12 weeks after initiating the treatment***		***Between-Group Difference in Mean Improvement***	
	Intervention group (IG)	*Control comparator group (CG)*	*Difference in LSMeans (95%CI)†*	*P-Value*
	LS Means (SE)†	LS Means (SE)†		
**Primary endpoint**				
Change SPADI overall score* (0 to 100)	-9.6 (2.7)	-14.5 (2.8)	-4.8 (-12.5 to 2.9)	0.22
**Key secondary outcome measures**				
Change SPADI pain* (0-100)	-12.2 (3.7)	-17.0 (3.8)	-4.7 (-15.4 to 6.0)	0.38
Change SPADI function* (0-100)	-7.2 (2.3)	-11.9 (2.4)	-4.7 (-11.3 to 1.9)	0.16

* SPADI ranges from 0 (best) to 100 (worst), with lower scores indicating better disease status

† Estimates are least squares means (LSMeans) and standard errors (SE) with the difference between groups reported with 95% confidence intervals (CI)
